# *Pseudomacrochenus wusuae* sp. n., a new species from Sichuan, China (Coleoptera, Cerambycidae, Lamiinae)

**DOI:** 10.3897/zookeys.656.11676

**Published:** 2017-02-14

**Authors:** Li He, Bin Liu, Cheng-Bin Wang

**Affiliations:** 1 State Grid Tianfu New Area Electric Power Supply Company, Chengdu 610094, P. R. China; 2 Bin Insect Taxonomy Studio, No.16, Xizhaosi Street, Dongcheng District, Beijing 100061, P. R. China; 3 Department of Ecology, Faculty of Environmental Sciences, Czech University of Life Sciences Prague, Kamýcká 129, CZ-165 21 Praha 6, Czech Republic

**Keywords:** Cerambycidae, China, Lamiinae, Lamiini, new species, *Pseudomacrochenus*, taxonomy

## Abstract

*Pseudomacrochenus wusuae* **sp. n.** (Coleoptera, Cerambycidae, Lamiinae, Lamiini) is described from Sichuan, China. Relevant morphological characters are illustrated by colour plates and a differential diagnosis of the new species from its relatives is provided.

## Introduction

The Oriental genus *Pseudomacrochenus*, belonging to the tribe Lamiini in the subfamily Lamiinae (Coleoptera: Cerambycidae), was originally established by Breuning (1943), with *Pelargoderus antennatus* Gahan, 1894 as the type species fixed by the original designation.

*Pseudomacrochenus* Breuning, 1943 is a small genus only composed of five valid species: *Pseudomacrochenus antennatus* (Gahan, 1894), *Pseudomacrochenus spinicollis* Breuning, 1949, *Pseudomacrochenus oberthueri* Breuning, 1955, *Pseudomacrochenus affinis* Breuning, 1960, *Pseudomacrochenus albipennis* Chiang, 1981; their geographical distributions are generally limited to the northern Oriental region. Undoubtedly, China is the distribution center of *Pseudomacrochenus* since four species were recorded and two of them are endemic to China (Wang 1997, Löbl & Smetana 2010). In this paper, a new species is described: *Pseudomacrochenus wusuae* sp. n., which was collected from Xichang City, Sichuan Province, China.

## Material and methods

Specimens were relaxed and softened in a hot saturated solution of potassium hydroxide for three minutes, and then transferred into distilled water to rinse the residual potassium hydroxide off and stop any further bleaching. The softened specimens were moved into glycerin and dissected there to observe morphological details. After examination, the body parts were mounted on plastic slips with gum arabic for future studies. Habitus photographs were taken using a Canon 50D DSLR with EF 100mm f/2.8L IS USM lens. Observations, photographs and measurements of morphological details were performed using a Zeiss Axio Zoom V16 motorized stereo zoom microscope (magnification up to ×270) with a Zeiss AxioCam MRc 5. The final deep focus images were created with Helicon Focus 5.3 or Zerene Stacker 1.04 stacking softwares. Adobe Photoshop® CS6 was used for post processing. Measurements are averaged over five specimens.

Relevant morphological characters are illustrated with colour plates and a differential diagnosis of the new species from its relatives is provided.

The material examined for this study is deposited in the following collections and museums:

BITS Bin Insect Taxonomy Studio, Beijing, China

CCWI Collection of Wen-I Chou, Taitung, Taiwan, China

CCZC Collection of Chao Zhou, Chengdu, Sichuan, China

CJYT Collection of Junsuke Yamasako, Tokyo, Japan

CLHC Collection of Li He, Chengdu, Sichuan, China

NMPC Národní museum, Prague, Czech Republic

## Results

### Genus Pseudomacrochenus

Taxon classificationAnimaliaColeopteraCerambycidae

Breuning, 1943

Vernacular name: 伪鹿天牛属

#### Distribution.

North Oriental.

### Pseudomacrochenus wusuae sp. n.

Taxon classificationAnimaliaColeopteraCerambycidae

http://zoobank.org/C9924F5B-C4EE-4B95-BD8F-324CCA00FF89

Vernacular name: 午苏伪鹿天牛

**Figure 1.**
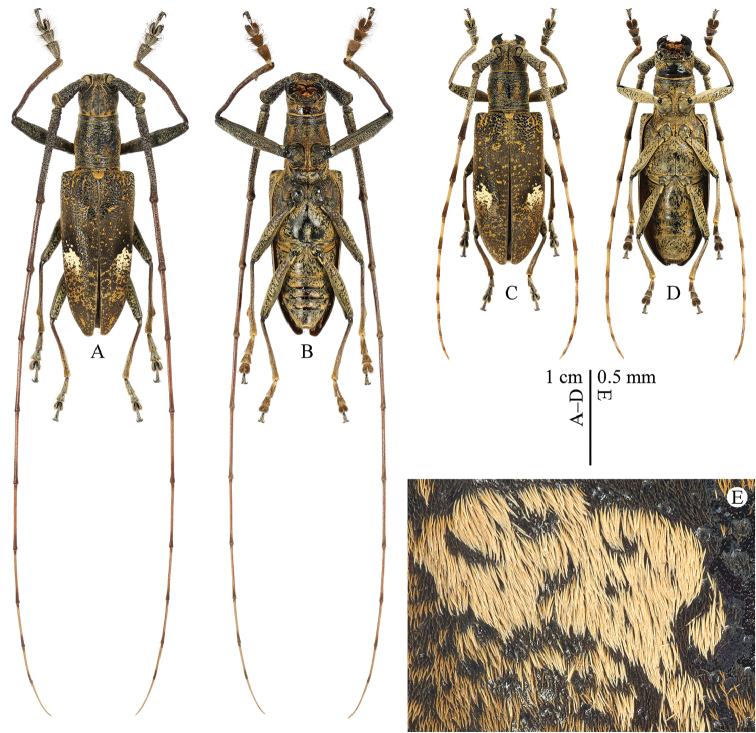
*Pseudomacrochenus wusuae* sp. n. **A** ♂ habitus (holotype; dorsal view) **B** ♂ habitus (holotype; ventral view) **C** ♀ (paratype; dorsal view) **D** ♀ (paratype; ventral view) **E** magnification of a grayish large spot on the elytron (♂; paratype; dorsal view).

**Figure 2.**
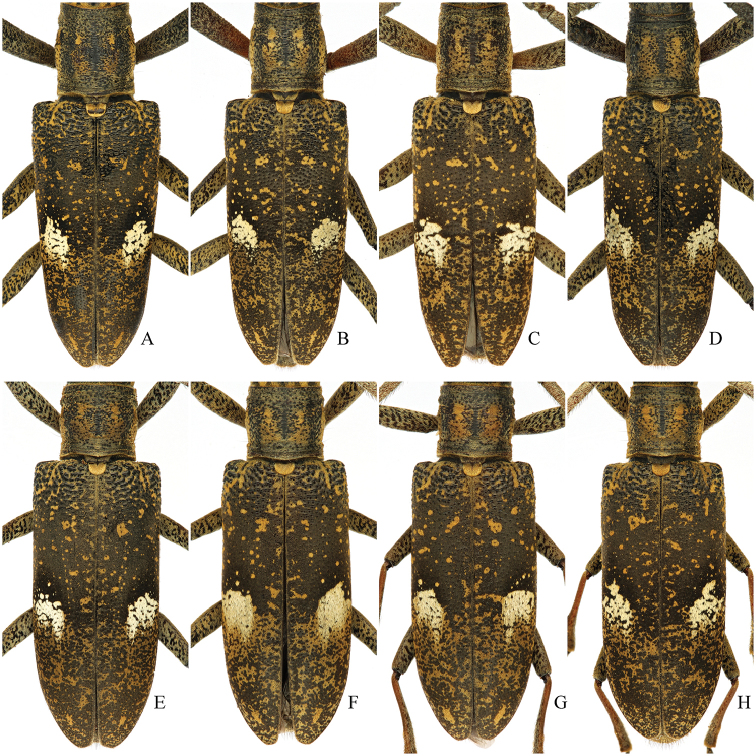
Variations of pubescence and spots on the elytra of *Pseudomacrochenus wusuae* sp. n. (paratypes; dorsal view) **A–D** ♂♂, **E–H** ♀♀.

**Figure 3.**
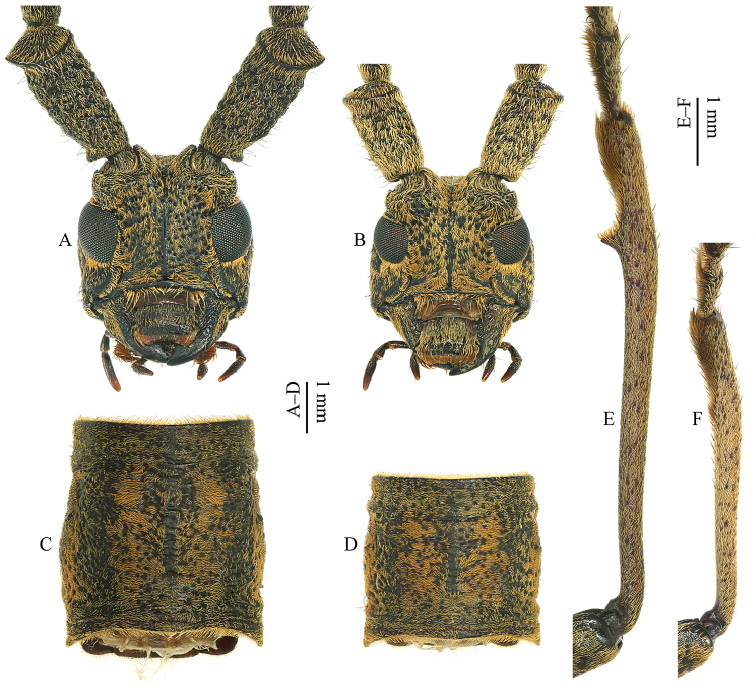
*Pseudomacrochenus wusuae* sp. n. (paratype). **A** head (♂; front view) **B** head (♀; front view) **C** pronotum (♂; dorsal view) **D** pronotum (♀; dorsal view) **E** protibia (♂; dorsal view) **F** protibia (♀; dorsal view).

**Figure 4.**
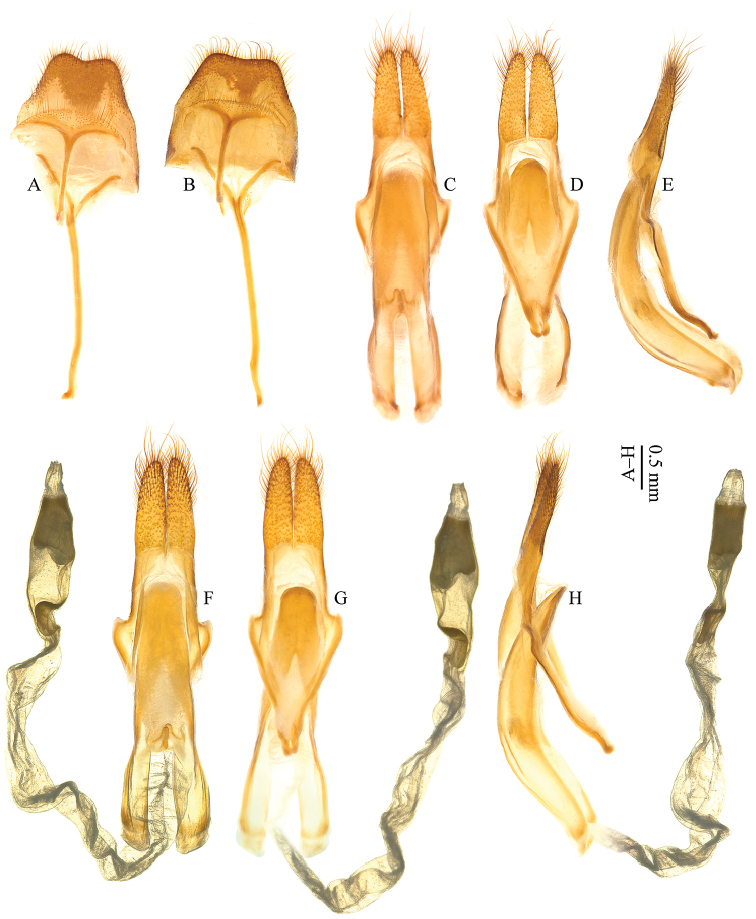
*Pseudomacrochenus* species. ♂♂. **A, C–E** *Pseudomacrochenus antennatus* (Gahan, 1894) (Yunnan) **B, F–H** *Pseudomacrochenus wusuae* sp. n. (paratype) **A–B** tergites VIII, sternites VIII & IX (ventral view) **C–H** male genitalia (**C, F** dorsal view **D, G** ventral view **E, H** lateral view).

**Figure 5.**
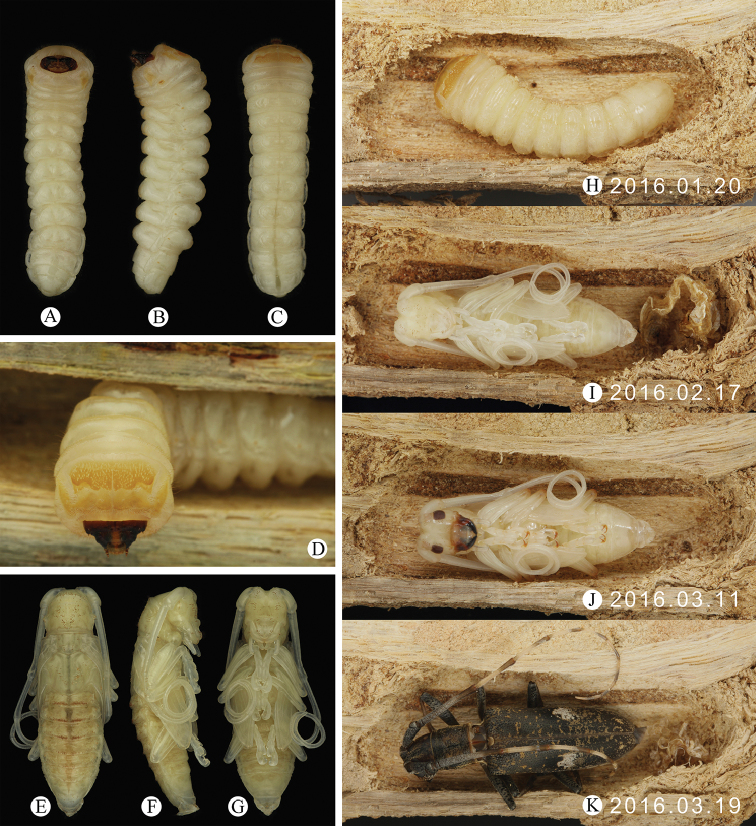
*Pseudomacrochenus wusuae* sp. n. (paratypes). **A–D** last instar larva (**A** ventral view **B** ventrolateral view **C** dorsal view **D** front view) **E–G** pupa (**E** dorsal view **F** lateral view **G** ventral view) **H** last instar larva in pupal chamber (20.I.2016) **I** pupa in pupal chamber (17.II.2016) **J** pupa in pupal chamber (11.III.2016) **K** newly sclerotized adult in pupal chamber (19.III.2016).

**Figure 6.**
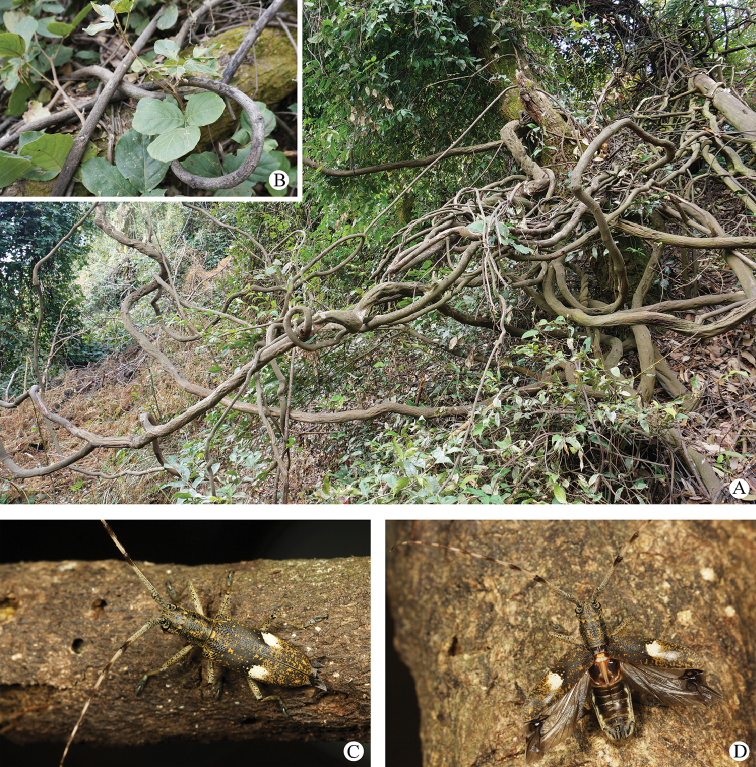
Field observations of *Pseudomacrochenus wusuae* sp. n. **A** biotope **B** host plant *Craspedolobium schochii* Harms **C** adult (resting) **D** adult (preparing to fly).

Figs 1 , 2 , 3 , 4B, F–H , 5 , 6C–D

#### Type material.

**Holotype**: ♂, CHINA, Sichuan: Liangshan Yi Autonomous Prefecture, Xichang City, Mt. Lushan (泸山) N27°49', E102°15', alt. 2050 m, 8.V.2015, Li He leg. (BITS); **Paratype**: 23♂♂39♀♀. 5♂♂4♀♀, same data as holotype except 16–17.XI.2015 (larva), em. II–III.2016, Li He & Bin Liu leg. (5♂♂ in NMPC and 4♀♀ in CLHC); 2♀♀, same data as holotype except alt. 2230 m, 8.VIII.2012, Li He leg. (CLHC); 1♀, same data as holotype (CLHC); 1♀, same data as holotype except 16–17.XI.2015 (larva), 17.II.2016 (pupa), em. 16.III.2016, Li He & Bin Liu leg. (CLHC); 1♀, same data as holotype except 16.XI.2015 (larva), em. 3.III.2016, Li He & Bin Liu leg. (BITS); 1♂, same data as holotype except alt. 2095 m, 17.XI.2015 (larva), em. 16.II.2016, Li He & Bin Liu leg. (BITS); 2♀♀, same data as holotype except 5.III.2016 (larva), em. 3.IV.2016, Li He leg. (CLHC); 7♂♂7♀♀, same data as holotype except 7.V.2016, Li He & Ben-Fu Miao leg. (CLHC); 1♂, same data as holotype except alt. 1928 m, 11.VI.2016, Bin Liu leg. (BITS); 2♂♂7♀♀, same data as holotype except alt. 1900 m, 11–16.VI.2016, Bin Liu leg. (CJYT); 1♂, same data as holotype except alt. ca. 2000 m, 12.VI.2015, Bin Liu leg. (CJYT); 1♂1♀, same data as holotype except alt. 1900 m, 11–16.VI.2016, Bin Liu leg. (CCWI); 1♂2♀♀, same data as holotype except alt. 1928 m, 12.VI.2016, Bin Liu leg. (BITS); 2♀♀, same data as holotype except alt. 2040 m, 12.VI.2016, Bin Liu leg. (BITS); 1♀, same data as holotype except alt. 1928 m,13.VI.2016, Bin Liu leg. (BITS); 5♀♀, same data as holotype except alt. 2049 m, 16.VI.2016, Bin Liu leg. (BITS); 1♂, same data as holotype except alt. 1703 m, 20.VI.2016, Bin Liu leg. (BITS); 1♀, same data as holotype except alt. 1928 m, 20.VI.2016, Bin Liu leg. (BITS); 1♀, same data as holotype except alt. 1647 m, 21.VI.2016, Bin Liu leg. (BITS); 1♂1♀, same data as holotype except 25.VI.2016, Chao Zhou, Bin Liu & Li He leg. (CCZC); 1♂, same data as holotype except 25.VI.2016, Chao Zhou, Bin Liu & Li He leg. (BITS).

#### Diagnosis.

Pronotum without spine at the lateral side, but only with an inconspicuous vestigial small tubercle. Elytra with a contrasting large spot on the middle constituted of pale grayish setae, except for a hairless area around the anterior margin forming a black semicircular ring. Abdominal tergite VIII with posterior edge weakly emarginate; sternite VIII short, with posterior edge more or less truncate.

#### Description.

**Male.** Size relatively large, body length 16.22–30.20 mm, humeral width 4.48–8.72 mm. Length (mm) of different body parts: head (3.53) : antenna (68.27) : pronotum (5.37) : elytra (17.39) : protibia (7.57); width (mm): head (3.44) : pronotum (4.91) : elytra (7.84). Body length/elytral width = 3.50; antenna length/body length = 2.49. Antennomeres with length ratio from base to tip: 6.70 - 1.00 - 16.38 - 12.29 - 12.33 - 12.80 - 12.92 - 11.78 - 9.70 - 7.85 - 13.64.

Habitus is shown in Fig. 1A–B. Body color dark brown to black. Head covered with fulvous setae, forming four small spots at posterior margin of the occiput. Dorsal surface of scape, pedicel and antennomere III with fulvous setae, following antennomeres with very faint fulvous setae; apical parts of the antennomeres III–X and middle part of the antennomere XI with very faint brown setae, making alternant contrasting colors on these antennomeres; ventral surface of scape, pedicel and antennomeres III–IV fringed with long, brown setae, but distinctly less dense on the antennomere IV. Pronotum with middle line flanked by two ill-defined longitudinal fasciae of fulvous setae and each pronotal side with another one. Elytra mostly covered with very faint brown setae; a contrasting large spot on the middle constituted of pale grayish setae (Fig. 1E), except for a hairless area around the anterior margin forming a black semicircular ring; a number of irregularly scattered small spots of fulvous setae forming three or four short longitudinal fasciae contiguous at base and much denser and contiguous after the discal spot. Variations of pubescence is shown in Fig. 2A–D. Abdomen covered with fulvous setae, lateral margins with some erected brown setae.

Head (Fig. 3A) compressed, surface coarsely granulated, length/width = 1.03, narrower than pronotum. Frons transverse, slightly convex. Eyes small, finely facetted, divided into two widely separated lobes; lower lobes longer than genae. Interantennal region strongly concave between the strongly elevated antennal tubercles. Antennae long, extending beyond elytral apex by six antennomeres; scape stout and cylindrical, larger at apex, with an open circular scar; surface of scape, pedicel and antennomere III with small and coarse granules; antennomere III longer than all others; antennomeres IV, V, VI, VII and XI subequal; antennomeres VII to X decreasing in length; last antennomere thinner and curved.

Pronotum (Fig. 3C) slightly convex and elongate, length/width = 1.09, widest at about basal 3/7 where it is slightly protruded; anterior margin slightly concave; lateral side with an inconspicuous vestigial small tubercle; hind angles slightly projected backwards and somewhat acute; surface coarsely rugose.

Stridulatory organ hided with a median longitudinal band of dense, fine, transverse stridulatory striae.

Scutellum ligulate, surface densely covered with fulvous setae.

Elytra widest just after humeri, length/width = 2.22, gradually narrowing towards apex; apices narrowly rounded; surface with many small and coarse granules at base, more or less thickly punctured with punctures diminishing in size towards apex.

Metathoracic wings fully developed.

Prolegs elongated; protibia (Fig. 3E) slightly sinuate, with a strong tooth at about the apical fourth of inner side. Profemora longer than meso- and metafemora. Metafemora exceed the posterior edge of visible abdominal segment IV.

Abdomen (Fig. 4B): tergite VIII with posterior edge weakly emarginate, bordered with long setae; sternite VIII short, with posterior edge more or less truncate, bordered with much shorter setae; sternite IX ‘Y’-shaped.

Male genitalia (Fig. 4F–H): Lateral lobes of tegmen moderately elongate, gradually tapering to narrowly rounded apex, which carries long setae. Median lobe stout, median struts more than half length of median lobe; ventral plate longer than dorsal plate; ventral plate with apex widely rounded. Endophallus with tubular structure at basal end.

**Female.** Size smaller than male, body length 16.67–25.47 mm, humeral width 4.37–7.55 mm. Length (mm) of different body parts: head (3.49) : antenna (32.89) : pronotum (3.92) : elytra (15.84) : protibia (4.65); width (mm): head (3.20) : pronotum (4.51) : elytra (7.45). Body length/elytral width = 3.13; antenna length/body length = 1.41. Antennomeres with length ratio from base to tip: 6.03 - 1.00 - 14.07 - 9.51 - 8.10 - 7.03 - 6.33 - 5.02 - 4.12 - 3.71 - 5.56.

Habitus is shown in Fig. 1C–D. Eyes (Fig. 3B) with lower lobe shorter than genae. Antennae shortened, extending beyond the elytral apex by five antennomeres. Pronotum (Fig. 3D) slightly wider than long, length/width = 0.87, widest at middle. Variations of pubescence and spots on the elytra is shown in Fig. 2E–H. Protibiae (Fig. 3F) not elongated, without observable tooth at inner side.

#### Immature stages.

Some logs containing larvae were chopped from the type locality and then transferred to the laboratories of Chengdu and Beijing in a constant temperature of 25°C. By observing the pupal chamber (Fig. 5H–K) every day, we observed that *Pseudomacrochenus wusuae* sp. n. took about 28 days from last instar larva (20.I.2016) to pupa (17.II.2016) and about 31 days from pupa to emergence. The habitus of last instar larva is shown in Fig. 5A–D and pupa is shown in Fig. 5E–G.

#### Host plant.

*Craspedolobium schochii* Harms (巴豆藤) (Fig. 6A–B).

**Field observations.** Biotope in broad-leaved mixed forest of Liangshan Yi Autonomous Prefecture (Sichuan) is shown in Figs 6A–B. Adults in the biotope are shown in Fig. 6C–D.

#### Remarks.

It is easy to distinguish *Pseudomacrochenus wusuae* sp. n. from *Pseudomacrochenus spinicollis* Breuning, 1949, *Pseudomacrochenus oberthueri* Breuning, 1955 and *Pseudomacrochenus albipennis* Chiang, 1981 since the new species has pronotum (Fig. 3C–D) much longer than wide, without long spine at the lateral side, but only with an inconspicuous vestigial small tubercle; while the latter three species have pronotum less elongated, with a distinct spine at each lateral side. In addition, pubescence and spots on the elytra of these species are different.

This new species well resembles *Pseudomacrochenus antennatus* (Gahan, 1894) in general appearance but it is easily distinguishable from it by the combination of the following characters: in *Pseudomacrochenus wusuae* sp. n., elytra with a large discal spot constituted of pale contrasting grayish setae (Fig. 1E); area around the anterior margin of this spot almost not pubescent, forming a black semicircular ring; tergite VIII (Fig. 4B) with posterior edge weakly emarginate; sternite VIII (Fig. 4B) short and posterior edge more or less truncate; lateral lobes (Fig. 4F) of tegmen with more setae on dorsal surface of apex; ventral plate with apex (Fig. 4G) widely rounded. In *Pseudomacrochenus antennatus*, elytra without contrasting large spot; tergite VIII (Fig. 4A) with posterior edge distinctly emarginate; sternite VIII (Fig. 4A) longer and posterior edge roundly curved; lateral lobes (Fig. 4C) of tegmen with less setae on dorsal surface of apex; ventral plate with apex (Fig. 4D) rounded.

This new species is also similar to *Pseudomacrochenus affinis* Breuning, 1960, from which it can be distinguished due to the fact that *Pseudomacrochenus wusuae* sp. n. shows a larger discal spot, with less defined borders, and a hairless black semicircular ring around the anterior margin; while *Pseudomacrochenus affinis* shows a smaller discal spot, with sharply defined borders, and a quite large hairless black patch before the eytral apex.

#### Etymology.

The specific epithet is dedicated to Ms. Wu-Su Chen, the wife of the first author, for her constant support and love.

#### Distribution.

China (Sichuan).

## Supplementary Material

XML Treatment for Pseudomacrochenus

XML Treatment for Pseudomacrochenus wusuae
